# Klotho-derived peptide KP1 ameliorates SARS-CoV-2-associated acute kidney injury

**DOI:** 10.3389/fphar.2023.1333389

**Published:** 2024-01-03

**Authors:** Jie Xu, Enqing Lin, Xue Hong, Li Li, Jun Gu, Jinghong Zhao, Youhua Liu

**Affiliations:** ^1^ State Key Laboratory of Organ Failure Research, Division of Nephrology, Nanfang Hospital, Southern Medical University, Guangzhou, China; ^2^ National Clinical Research Center of Kidney Disease, Guangdong Provincial Institute of Nephrology, Guangzhou, China; ^3^ State Key Laboratory of Protein and Plant Gene Research, College of Life Science, Peking University, Beijing, China; ^4^ Division of Nephrology, Xinqiao Hospital, Army Medical University, Chongqing, China

**Keywords:** COVID-19, acute kidney injury, aging, Klotho, Klotho-derived peptide

## Abstract

**Introduction:** The severe cases of COVID-19, a disease caused by severe acute respiratory syndrome coronavirus-2 (SARS-CoV-2), often present with acute kidney injury (AKI). Although old age and preexisting medical conditions have been identified as principal risk factors for COVID-19-associated AKI, the molecular basis behind such a connection remains unknown. In this study, we investigated the pathogenic role of Klotho deficiency in COVID-19-associated AKI and explored the therapeutic potential of Klotho-derived peptide 1 (KP1).

**Methods:** We assessed the susceptibility of Klotho deficient *Kl/Kl* mice to developing AKI after expression of SARS-CoV-2 N protein. The role of KP1 in ameliorating tubular injury was investigated by using cultured proximal tubular cells (HK-2) *in vitro* and mouse model of ischemia-reperfusion injury (IRI) *in vivo*.

**Results:** Renal Klotho expression was markedly downregulated in various chronic kidney disease (CKD) models and in aged mice. Compared to wild-type counterparts, mutant *KL/KL* mice were susceptible to overexpression of SARS-CoV-2 N protein and developed kidney lesions resembling AKI. *In vitro*, expression of N protein alone induced HK-2 cells to express markers of tubular injury, cellular senescence, apoptosis and epithelial-mesenchymal transition, whereas both KP1 and Klotho abolished these lesions. Furthermore, KP1 mitigated kidney dysfunction, alleviated tubular injury and inhibited apoptosis in AKI model induced by IRI and N protein.

**Conclusion:** These findings suggest that Klotho deficiency is a key determinant of developing COVID-19-associated AKI. As such, KP1, a small peptide recapitulating Klotho function, could be an effective therapeutic for alleviating AKI in COVID-19 patients.

## 1 Introduction

Coronavirus disease 2019 (COVID-19) is a disease caused by severe acute respiratory syndrome coronavirus-2 (SARS-CoV-2). According to the estimate by World Health Organization, COVID-19 epidemic caused more than 7 million deaths worldwide, most of them are the elderly and those with preexisting medical conditions such as diabetes mellitus, hypertension, chronic kidney disease (CKD) and cardiovascular disease (Grasselli et al., 2020; Parohan et al., 2020; Zhou et al., 2020). The severe cases of COVID-19 are frequently manifested with acute kidney injury (AKI), affecting 32.6%–86.1% of intensive care unit COVID-19 patients (Chan et al., 2021; Figueiredo et al., 2022; Geetha et al., 2022; Hsu et al., 2022; Schaubroeck et al., 2022). The severity of AKI is closely associated with in-hospital mortality and poor outcomes (Gu et al., 2022; Long et al., 2022). Although old age and preexisting medical condition have been identified as the principal risk factors for COVID-19-associated AKI, the molecular basis behind such a connection remains largely enigmatic.

The α-Klotho, referred as Klotho hereafter, is an anti-aging protein that is highly expressed in the tubular epithelial cells of normal kidney (Kuro-o et al., 1997). The *Kl/Kl* mice with mutations in the *Klotho* gene exhibit complete deficiency of Klotho protein and have the phenotype of premature aging (Kuro-o et al., 1997). Klotho protein is composed of two large homologous KL1 and KL2 domains in the extracellular region, a transmembrane segment and a short cytoplasmic tail. Besides this full-length membranous form, alternative splicing and/or proteolytic shedding also generate soluble Klotho, which is released into the circulation and functions as a hormone (Hu et al., 2012; Chen et al., 2014). The levels of Klotho in serum and kidney tissues are decreased in the elderly, as well as in AKI, CKD, diabetes and cardiovascular disease patients with diverse etiologies (Lin et al., 2013; Zhou et al., 2013; Fu and Liu, 2015; Kim et al., 2016; Fountoulakis et al., 2018; Neyra et al., 2020). These findings underscore that loss of Klotho is a common characteristic feature shared by the elderly and those with various preexisting medical conditions. Whether Klotho deficiency plays a role in the pathogenesis of AKI in COVID-19 patients, however, is unknown.

SARS-CoV-2 virion contains four major structural proteins: spike (S), envelope (E), membrane (M), and nucleocapsid (N). While it is widely accepted that the S protein plays a crucial role in COVID-19 infection by mediating the SARS-CoV-2 entry into the host cells, studies have shown that N protein is obligatory and essential for actually causing cell injury via multiple signaling pathways, leading to activation of NLRP3 inflammation, TGF-β/Smad3 signaling, cell cycle arrest and apoptosis (Pan et al., 2021; Wang et al., 2022). Although whether SARS-CoV-2 directly infects the kidney cells remains controversial, several studies have established the presence of SARS-CoV-2 N protein and RNA in the kidney of autopsy and biopsy specimens, respectively. Single-nucleus RNA sequencing (RNA-seq) also demonstrates that all 13 identified cell clusters in COVID-19 patient’s renal autopsy express SARS-CoV-2 RNA, particularly in the proximal tubular cells and podocytes (Hassler and Batlle, 2022; Jansen et al., 2022). Furthermore, recent studies provide compelling evidence that SARS-CoV-2 N protein triggers AKI via the Smad3-dependent mechanism (Wang et al., 2022). In view of that Klotho is kidney protective by inhibiting TGF-β signaling (Zhou et al., 2013), it is conceivable to speculate that Klotho deficiency in the elderly and those with preexisting medical condition would make them susceptible to cell injury and AKI caused by COVID-19 infection.

In this study, we demonstrated that Klotho deficiency in *Kl/Kl* mice sensitizes them to develop AKI after SARS-CoV-2 N protein expression. Furthermore, we showed that KP1, a Klotho-derived peptide with 30 amino acids that recapitulates the kidney protection of full-length Klotho, inhibits tubular cell apoptosis and ameliorates kidney dysfunction *in vitro* and *in vivo*. Our findings suggest that KP1, a short peptide that can be made chemically and cost-effectively, could become a new remedy for preventing and treating AKI in COVID-19 patients.

## 2 Materials and methods

### 2.1 Peptide synthesis and plasmid construction

The KP1 peptide with 30 amino acids was derived from human Klotho protein and described previously (Yuan et al., 2022). KP1 was synthesized by GenScript (Piscataway, NJ) with the purity of >95%. The peptide was dissolved in 0.01 M acetic acid at 10 μg/μL. Mammalian expression vector harboring the Flagged-tagged SARS-CoV-2 N Protein and empty vector pcDNA3 were constructed and prepared by GENEWIZ (Suzhou, China). The plasmids were purified by EndoFree Plasmid Maxi Kit (12263, QIAGEN, Germantown, MD).

### 2.2 Animal models

Male C57BL/6 mice were obtained from the Southern Medical University Animal Center (Guangzhou, China). Bilateral ischemia-reperfusion injury (IRI) was established as described (Luo et al., 2018). Briefly, mice were anesthetized using intraperitoneal injection of 1% pentobarbital sodium and bilateral renal pedicles were clipped for 28 min using microaneurysm clamps. During the ischemic period, body temperature was maintained at 37.5°C using a temperature-controlled heating system. After the removal of the clamps, reperfusion of the kidneys was visually confirmed. Kidney tissues and serum were collected at 2 days after IRI. For investigating the role of SARS-CoV-2 N protein in AKI, mice were injected with pSARS-CoV-2 N Protein (N) plasmid or empty vector pcDNA3 at a dose of 50 µg/mouse via tail vein 1 day before IRI. The KP1 peptide was dissolved in 0.01 M acetic acid and administrated intravenously via tail vein at the concentration of 1 mg/day/kg for 4 days, starting at 2 days prior to IRI. In addition, mouse models of unilateral ureteral obstruction (UUO), 5/6 nephrectomy (5/6Nx), db/db mice and their db/m controls (age at 20 weeks) and aged mice (24-months old) were used, as described previously (Zhou et al., 2015; Luo et al., 2018; Miao et al., 2019; Chen et al., 2022). Klotho-deficient mice (*Kl/Kl*) with C57BL/6J genetic background were described previously (Kuro-o et al., 1997). Briefly, WT and *Kl/Kl* mice were injected with pSARS-CoV-2 N Protein (N) or empty vector pcDNA3 at a dose of 50 µg/mouse via tail vein, respectively. All animal studies were approved by the Animal Ethics Committee at the Nanfang Hospital.

### 2.3 Serum creatinine and blood urea nitrogen assay

Serum creatinine (SCr) and blood urea nitrogen (BUN) were determined by an automatic chemistry analyzer. The levels of serum creatinine and BUN were expressed as mg/dL.

### 2.4 Cell culture and treatment

Human kidney proximal tubular cells (HK-2) were obtained from the American Type Culture Collection (ATCC; Manassas, VA). Briefly, HK-2 cells were grown in 6-well plates in DMEM/Ham’s F12 medium supplemented with 10% fetal bovine serum (FBS) and were cultured to be 60%–70% confluent at 37°C in an atmosphere containing 5% CO_2_ before transfection. Diluted pSARS-CoV-2 N Protein plasmid and Lipofectamine 2000 reagent were mixed in Opti-MEM Medium (Invitrogen, Grand Island, NY). The empty vector pcDNA3 (Invitrogen) was used as a negative control. Plasmid DNA-lipid complexes were added to the cells and incubated for 4–6 h. After transfection, cells were treated with KP1 (10 μg/mL) or recombinant human Klotho (100 ng/mL) (#5334-KL; R&D Systems) for 48 h. Cells were then collected and subjected to Western blot analyses or TUNEL staining, respectively.

### 2.5 TUNEL staining

TUNEL staining for apoptotic cells was performed according to the instructions of the DeadEnd Fluorometric TUNEL System (#G3250; Promega, Madison, WI). HK-2 cells, transfected with pSARS-CoV-2 N protein plasmid for 4–6 h and treated with KP1 or Klotho for 48 h, were fixed in 4% paraformaldehyde at room temperature. Cells were then incubated with rTdT incubation buffer containing equilibration buffer, nucleotide Mix and rTdT Enzyme in a water bath for 60 min at 37°C. Cell nuclei were stained with 4′,6-diamidino-2-phenylindole (DAPI). Samples were analyzed under a fluorescence microscope (Olympus, Tokyo, Japan) using a standard fluorescein filter set. Apoptosis was expressed as the percentage of TUNEL-positive cells to the total number of HK-2 cells.

### 2.6 Histology and immunohistochemical staining

Paraffin-embedded mouse kidney sections (3 µm thickness) were prepared by routine procedures. The sections were stained with periodic acid-Schiff (PAS) reagents by standard protocol. For immunohistochemical staining, kidney sections were deparaffinized with xylene, and then gradually rehydrated in ethanol. Hydrogen peroxide (3%) was used to eliminate endogenous peroxidase. Antigen retrieval was performed by microwave using the heat mediated antigen retrieval technique. After blocking with 1% donkey serum for 1 h, the sections were incubated with primary antibodies overnight at 4°C and subsequently washed and incubated with secondary antibodies for 1 h at room temperature. The sections were visualized by using AEC substrate Kit under an Olympus light microscope. The primary antibodies were as follows: anti-Coronavirus nucleocapsid (sc-66012; Santa Cruze Biotechnology), anti-KIM-1 (AF1817; R&D Systems), anti-p53 (A0263; Abclonal), anti-caspase-3 (A2156; Abclonal).

### 2.7 Western blot analysis

For preparing the protein sample, HK-2 cells and kidney tissues were lysed on ice using radioimmunoprecipitation assay (RIPA) lysis buffer, and the supernatants were collected after centrifugation at 13,000 g at 4°C for 15 min. Protein concentration was quantified using the BCA Protein Assay (K813-5000-1; BioVision). Proteins were separated with 8%–15% SDS-PAGE and blotted onto PVDF microporous membranes (IPVH00010; Millipore Sigma). The membranes were then blocked with 5% milk at room temperature for 1 h and incubated with primary antibodies at 4°C overnight, followed by incubating with HRP-conjugated secondary antibodies for 1 h at room temperature. The primary antibodies are as follows: anti-mKlotho (AF1819; R&D Systems), anti-PARP (#9532; Cell Signaling Technology), anti-p53 (#2524S; Cell Signaling Technology), anti-FADD (sc-271748; Santa Cruze Biotechnology), anti-cacspase-3 (#9622; Cell Signaling Technology), anti-Coronavirus nucleocapsid (sc-66012; Santa Cruze Biotechnology), anti-Flag (F1804; Sigma-Aidrich), anti-KIM-1 (Ab47635; Abcam), anti-Lipocalin-2/NGAL (ab63929; Abcam), anti-GAPDH (Z1208; Ray antibody Biotechnology). The protein bands were quantified by ImageJ software (NIH) and normalized to the loading controls.

### 2.8 Statistical analyses

All data examined were expressed as means ± SEM. Statistical analyses of the data were performed using SPSS 22.0. Comparison between groups was made by *t*-test when comparing two groups, or one-way analysis of variance (ANOVA) followed by Student-Newman-Kuels test for more than two groups. *p* < 0.05 was considered significant.

## 3 Results

### 3.1 Klotho deficiency is a common feature in mice with kidney disease or advanced age

**FIGURE 1:**
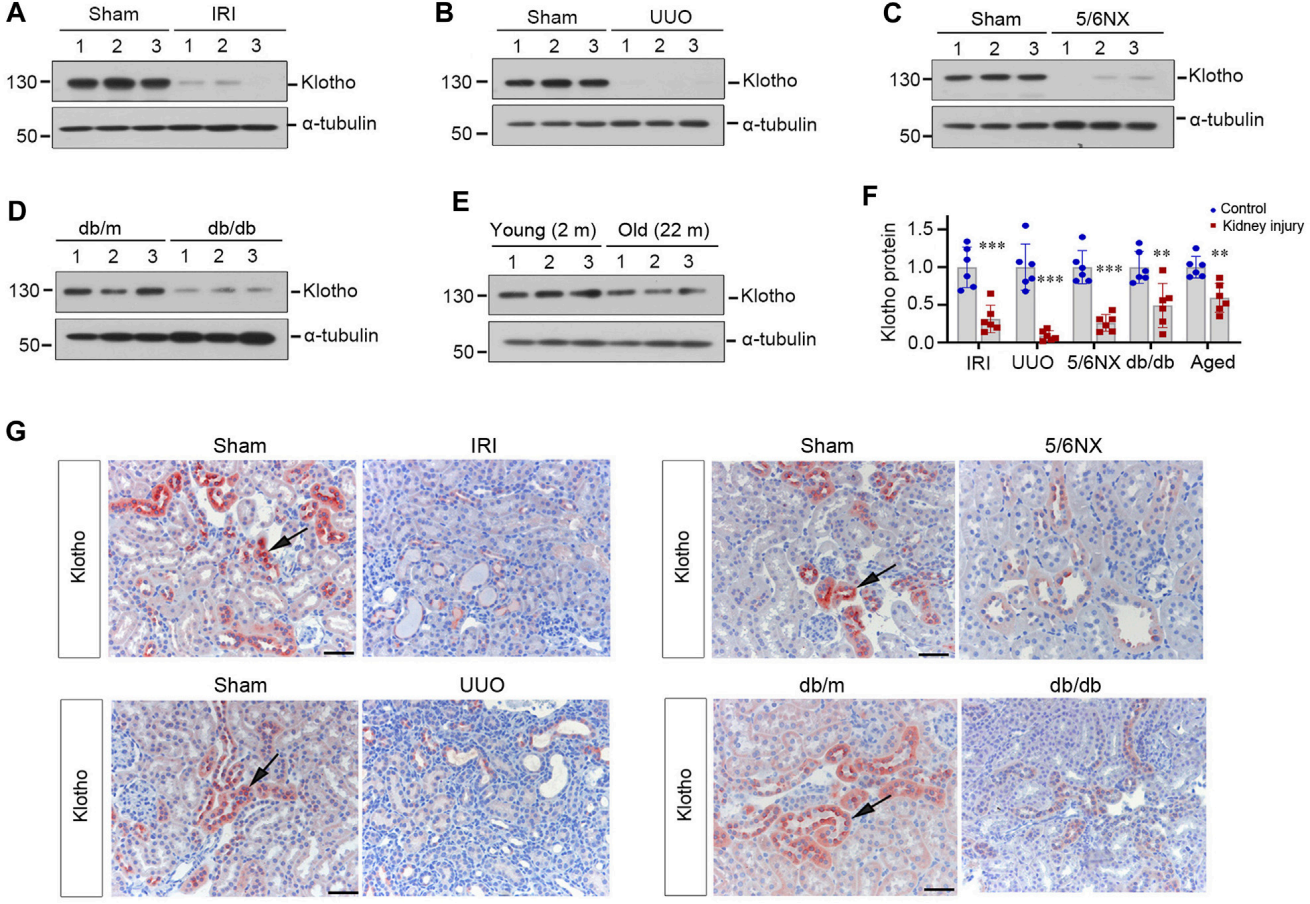
Klotho deficiency is a common feature of mouse kidney with preexisting chronic kidney diseases or advanced age. (**A**–**E**) Representative Western blots show renal protein level of Klotho in mouse models of ischemia-reperfusion injury (IRI), unilateral ureteral obstruction (UUO), remnant kidney after 5/6 nephrectomy (5/6Nx), db/db with diabetic kidney disease (DKD) and advanced age (24 months old), respectively. (**F**) Quantitative data of Klotho protein levels in the kidney of various models as indicated are presented. ^**^ *p* < 0.01 *versus* sham (*n* = 6). (**G**) Representative micrographs of immunochemical staining show Klotho expression and localization in different models of mice as indicated. Arrows indicate positive staining. Scale bar, 50 µm.

We first assessed the expression of Klotho protein in various animal models of nephropathies induced by IRI, UUO and 5/6NX, as well as in db/db or old mice, respectively. As shown in Figures 1A–F, renal Klotho protein was markedly reduced at 2 days after IRI, 7 days after UUO, 2 months after 5/6NX, 20-week old db/db mice and 22-month aged mice. These results suggest that Klotho deficiency is a common and shared feature in mice with kidney diseases or advanced age, which is consistent with previous findings in various animal models of CKD and in aged mice (Zhou et al., 2013; Chen et al., 2014; Zhou et al., 2015).

To confirm these observations, we next examined Klotho expression and localization in the kidney by using immunohistochemical staining. As shown in Figure 1G, Klotho protein was predominantly localized in renal tubular epithelium of normal kidneys. However, it was downregulated in diseased kidneys triggered by IRI, UUO, 5/6NX or hyperglycemia.

### 3.2 Klotho deficiency sensitizes mice to SARS-CoV-2 N protein-induced tubular injury

**FIGURE 2:**
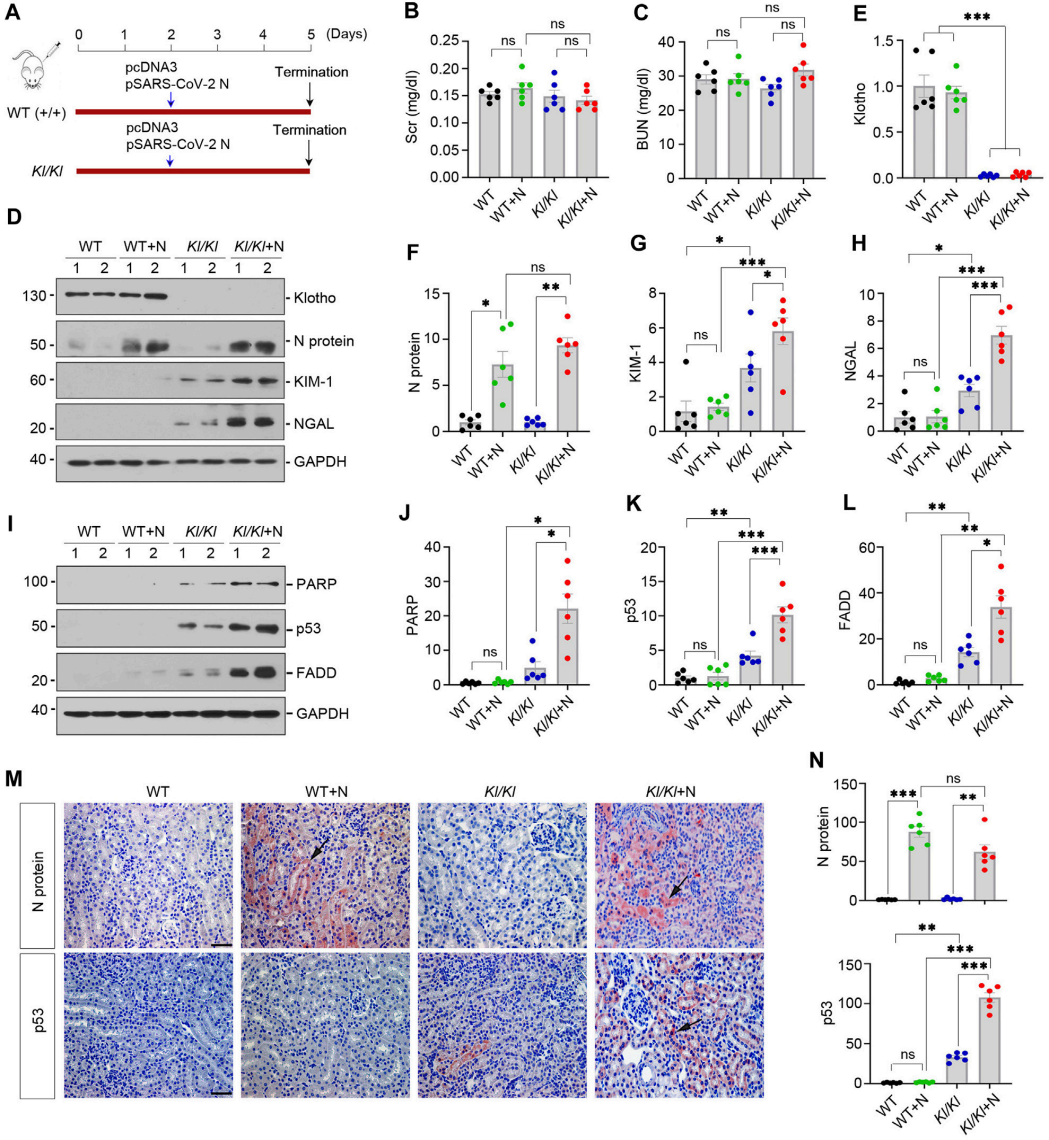
Klotho-deficiency sensitizes mice to SARS-CoV-2 N protein-triggered tubular injury and apoptosis. **(A)** Experimental design. The blue arrow indicates the timing of injecting pcDNA3 empty vector or pSARS-CoV-2 N Protein (N) plasmid into normal (WT) or Klotho-deficient (*KL/KL*) mice, respectively. **(B, C)** Graphic presentation shows serum creatinine (SCr) and blood urea nitrogen (BUN) levels in different groups. ns, not significant. **(D–H)** Representative Western blot **(D)** and quantitative data show renal protein levels of N Protein **(F)**, KIM-1 **(G)** and NGAL **(H)** in different groups. ^*^ *p* < 0.05, ^**^ *p* < 0.01, ^***^ *p* < 0.001 (*n* = 6). (**I**–**L**) Representative Western blot **(I)** and quantitative data show renal protein levels of cleaved PARP **(J)**, p53 **(K)** and FADD (**L**). ^*^ *p* < 0.05, ^**^ *p* < 0.01, ^***^ *p* < 0.001 (*n* = 6). (**M**) Representative micrographs of immunochemical staining show renal expression of N protein and P53 are presented. Arrows indicate positive staining. Scale bar, 50 µm. (**N**) Quantitative data of N Protein and p53. ^**^ *p* < 0.01, ^***^ *p* < 0.001 (*n* = 6).

As loss of Klotho is a common feature of various nephropathies or advanced age, this prompted us to hypothesize that a state of Klotho deficiency may sensitize mice to developing COVID-19-associated AKI. To test this hypothesis, we utilized *Kl/Kl* mice, in which Klotho protein is deficient due to gene mutation (Kuro-o et al., 1997). As shown in Figure 2A, wild-type (WT) and *Kl/Kl* mice were injected with pSARS-CoV-2 N plasmid or empty vector pcDNA3 by using the hydrodynamics-based gene delivery approach (Zhou et al., 2013). At 3 days after injection, we found that SARS-CoV-2 N protein did not induce kidney dysfunction, as the levels of SCr and BUN were unchanged in different groups as indicated (Figures 2B, C).

We next examined kidney injury-associated markers after N protein expression. As shown in Figures 2D–H, Western blot analysis of whole kidney lysates revealed that Klotho was undetectable in *Kl/Kl* mice and N protein was induced after injection of pSARS-CoV-2 N plasmid. In WT mice, kidney injury molecule-1 (KIM-1) and neutrophil gelatinase-associated lipocalin (NGAL), two widely used biomarkers for tubular injury, did not significantly change after N protein expression. Loss of Klotho in *Kl/Kl* mice caused a mild induction of KIM-1 and NGAL, compared with WT mice. However, overexpression of SARS-CoV-2 N protein markedly aggravated KIM-1 and NGAL induction in *Kl/Kl* mice, suggesting that loss of Klotho sensitizes mice to SARS-CoV-2 N protein-triggered tubular injury.

To further confirm tubular injury induced by N protein in Klotho-deficient mice, we examined the expression of apoptosis-related proteins such as cleaved poly-ADP-ribose polymerase (PARP), p53 and FAS-associated protein with death domain (FADD) in WT and *Kl/Kl* mice. In WT mice, overexpression of N protein did not significantly induce cleaved PARP, p53 and FADD (Figures 2I–L). However, in *Kl/Kl* mice, overexpression of N protein aggravated renal expression of cleaved-PARP, p53 and FADD (Figures 2I–L). As shown in Figure 2M, immunochemical staining revealed that N protein was primarily expressed in renal tubular epithelial cells after injection of pSARS-CoV-2 N plasmid. Consistently, p53 was only upregulated in *Kl/Kl* mice injected with pSARS-CoV-2 N plasmid (Figures 2M, N). Collectively, these results indicate that Klotho deficiency sensitizes kidney to tubular injury after SARS-CoV-2 N protein expression.

### 3.3 SARS-CoV-2 N protein induces cell injury and apoptosis *in vitro*

**FIGURE 3:**
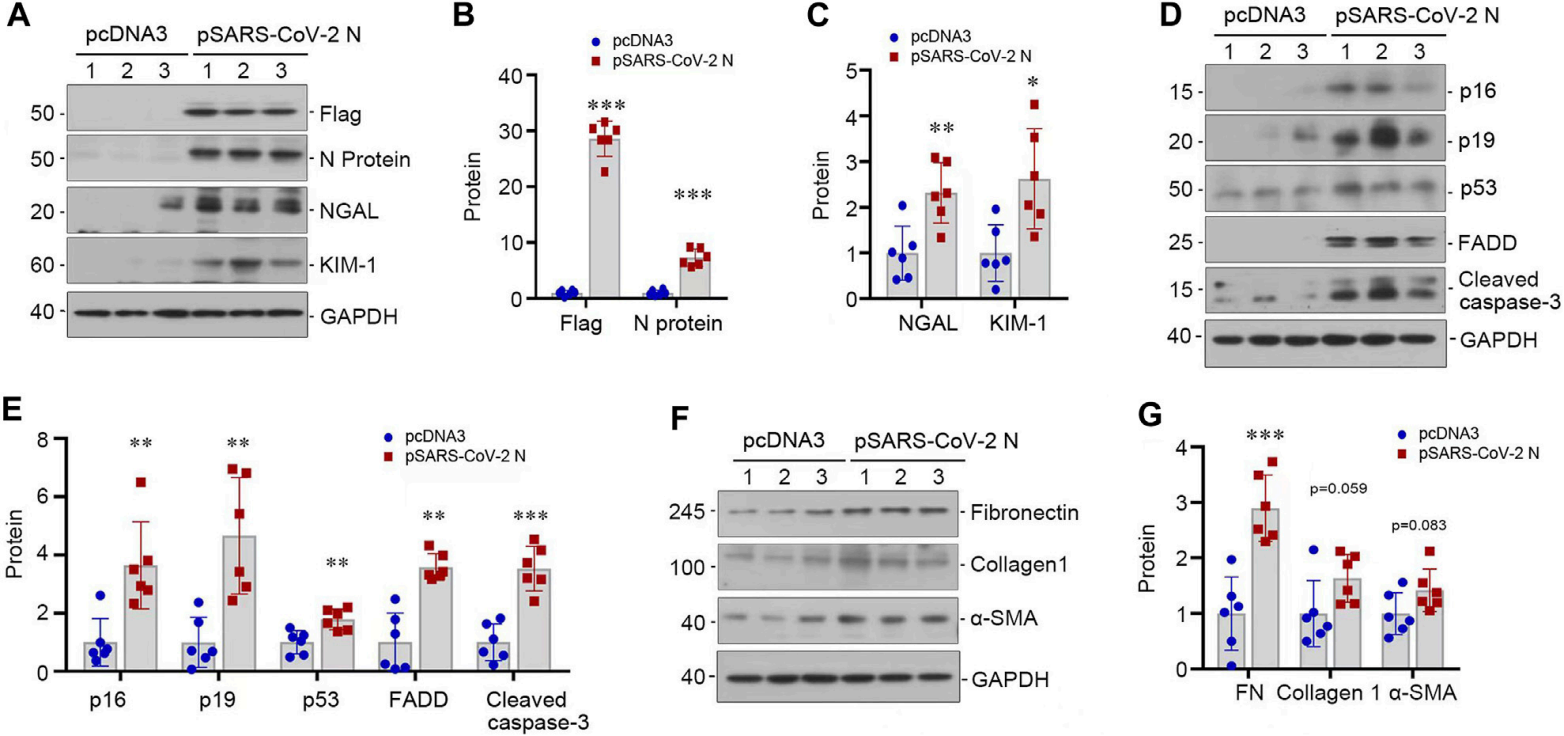
SARS-CoV-2 N protein induces tubular injury *in vitro*. **(A–C)** Representative Western blot **(A)** and quantitative data show the expression levels of Flag and N protein **(B)**, NGAL and KIM-1 **(C)** after transfecting SARS-CoV-2 N Protein plasmid (N) into HK-2 cells for 48 h. **(D, E)** Representative Western blot **(D)** and quantitative data show the expression levels of p16, p19, p53, FADD and cleaved caspase-3 **(E)** after transfecting SARS-CoV-2 N Protein plasmid (N) into HK-2 cells for 48 h. **(F, G)** Representative Western blot **(F)** and quantitative data show the expression levels of fibronectin, collagen 1, and α-SMA **(G)** after transfecting SARS-CoV-2 N Protein plasmid (N) into HK-2 cells for 48 h ^*^ *p* < 0.05, ^**^ *p* < 0.01, ^***^ *p* < 0.001 *versus* pcDNA3 (*n* = 6).

To validate the pathogenic role of SARS-CoV-2 N protein in renal tubular cells, we transfected HK-2 cells with pSARS-CoV-2 N plasmid or empty vector pcDNA3. As shown in Figures 3A, B, overexpression of Flag-tagged N protein was evident after transfection. We found that expression of N protein induced the expression of KIM-1 and NGAL (Figures 3A, C). Furthermore, N protein also upregulated the expression of senescent markers p16 and p19, as well as apoptosis-related proteins, including p53, FADD and cleaved caspase-3 (Figures 3D, E). In addition, overexpression of the N protein also caused the expression of several fibrosis-related proteins, such as fibronectin, collagen I and α-smooth muscle actin (α-SMA) (Figures 3F, G). These results indicated that the SARS-CoV-2 N protein directly triggers renal tubular cells injury and apoptosis *in vitro*.

### 3.4 KP1 ameliorates tubular cell injury and apoptosis induced by N protein *in vitro*

**FIGURE 4:**
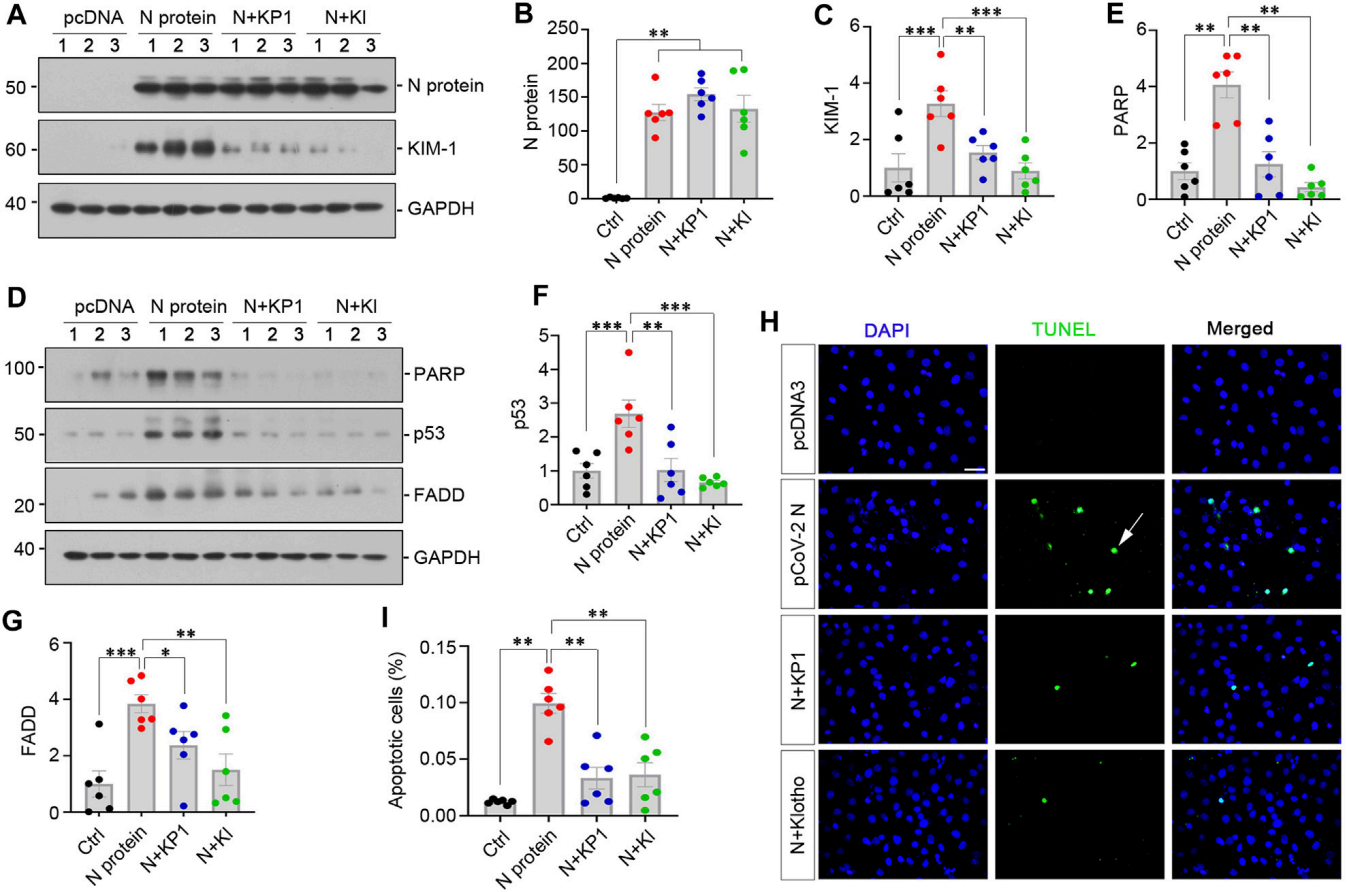
KP1 ameliorates tubular cell injury and apoptosis induced by SARS-CoV-2 N protein *in vitro*. **(A–C)** Representative Western blot **(A)** and quantitative data show the expression of N Protein **(B)** and KIM-1 **(C)**. Cells were treated with KP1 or recombinant human Klotho for 48 h after transfecting SARS-CoV-2 N Protein plasmid. ^**^ *p* < 0.01, ^***^ *p* < 0.001 (*n* = 6). **(D–G)** Representative Western blot **(D)** and quantitative data show the expression of PARP **(E)**, p53 **(F)** and FADD **(G)**. ^*^ *p* < 0.05, ^**^ *p* < 0.01, ^***^ *p* < 0.001 (*n* = 6). **(H, I)** Representative TUNEL staining **(H)** and quantitative data **(I)** show that SARS-CoV-2 N protein induced HK-2 cell apoptosis, while KP1 or Klotho (KL) protected HK-2 cells from apoptosis. ^**^ *p* < 0.01 (*n* = 6). Arrow indicates apoptotic cell.

We recently reported the discovery of KP1, a Klotho-derived peptide that exhibits kidney protective potential (Yuan et al., 2022; Zhang et al., 2024). This prompted us to test whether KP1 can prevent kidney tubular cells from injury caused by SARS-CoV-2 N protein. To this end, we treated HK-2 with KP1 or recombinant human Klotho after transfection with pSARS-CoV-2 N plasmid. As shown in Figures 4A, B, transfection with pSARS-CoV-2 N plasmid resulted in Flag-tagged N protein expression in HK-2 cells. Overexpression of N protein induced KIM-1, which was abolished by KP1 and Klotho (Figures 4A, C), suggesting that KP1 can imitate Klotho protein and ameliorates tubular damage caused by SARS-CoV-2 N protein. Consistently, overexpression of N protein increased the expression of cleaved PARP, p53 and FADD, which was abolished by KP1 as well (Figures 4D–G). Similar results were obtained when apoptotic cells were detected by TUNEL staining. As shown in Figures 4H, I, N protein increased tubular cell apoptosis, which was negated by KP1 or Klotho. Notably, the efficacy of KP1 in preventing tubular cell injury and apoptosis was comparable to Klotho itself (Figures 4A–I). These results suggest that KP1 can protect tubular epithelial cells against injury triggered by SARS-CoV-2 N protein.

### 3.5 KP1 ameliorates SARS-Cov-2 N-mediated AKI *in vivo*

**FIGURE 5:**
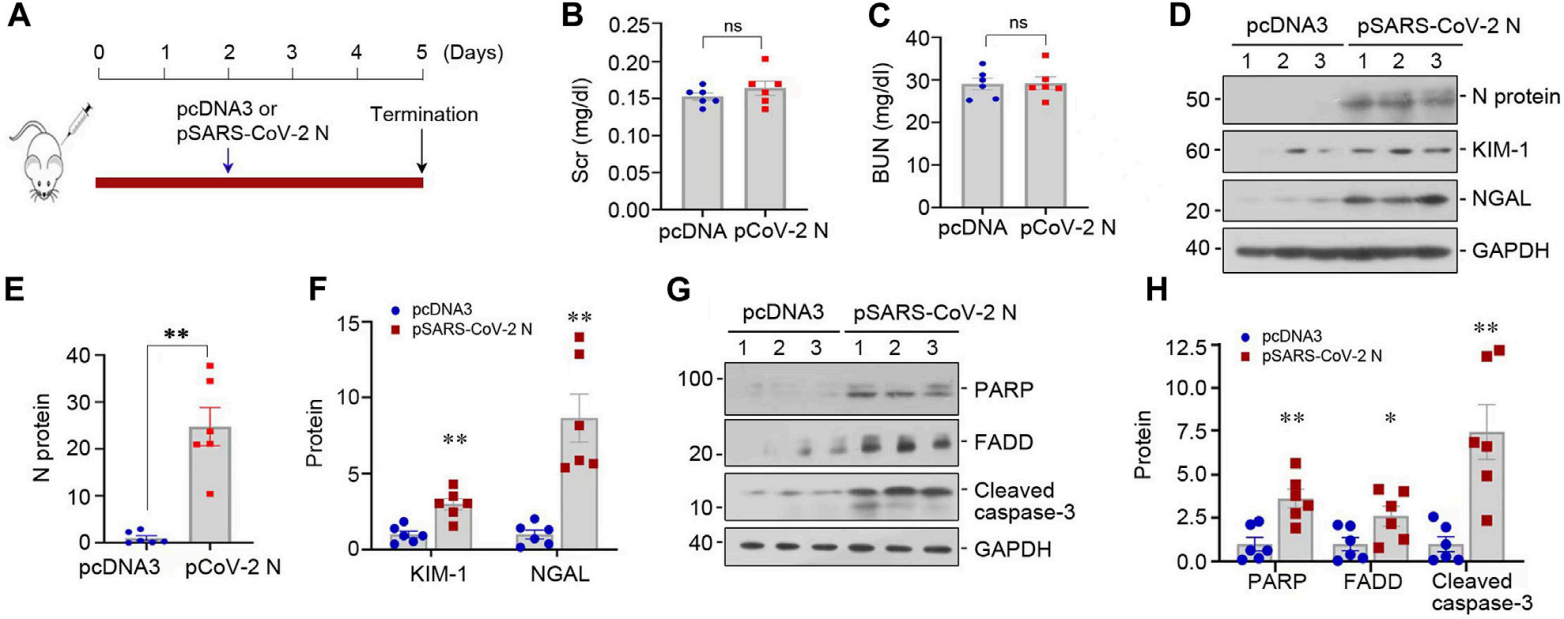
SARS-CoV-2 N protein plasmid alone does not cause kidney functional decline but triggers tubular cell injury *in vivo*. **(A)** Experimental design. The blue arrow indicates the timing of injecting pcDNA3 empty vector or pSARS-CoV-2 N Protein plasmid (N) into mice. **(B, C)** Graphic presentation shows SCr and BUN levels in different groups. ns, not significant (*n* = 6). **(D–F)** Representative Western blot **(D)** and quantitative data show renal protein levels of N protein **(E)**, KIM-1 and NGAL **(F)**. **(G, H)** Representative Western blot **(G)** and quantitative data show renal protein levels of PARP, FADD and cleaved caspase-3 **(H)**. ^*^ *p* < 0.05, ^**^ *p* < 0.01, ^***^ *p* < 0.001 *versus* pcDNA3 (*n* = 6).

We assessed the pathogenic role of SARS-CoV-2 N protein *in vivo*. As shown by Figure 5A, mice were injected with low dose of pSARS-CoV-2 N plasmid (50 µg/mouse) or empty control vector pcDNA3 and sacrificed at 3 days after injection. Although SCr and BUN levels did not change after overexpressing N protein in normal mice (Figures 5B, C), overexpression of N protein slightly induced the upregulation of KIM-1 and NGAL (Figures 5D–F). Consistently, the expression of apoptosis-related proteins was also increased in the kidney of mice injected with pSARS-CoV-2 N plasmid (Figures 5G, H). These observations suggest that low dose of SARS-CoV-2 N protein alone does not cause an appreciable decline in kidney function but triggers a slight induction of various injurious markers in normal mice.

**FIGURE 6:**
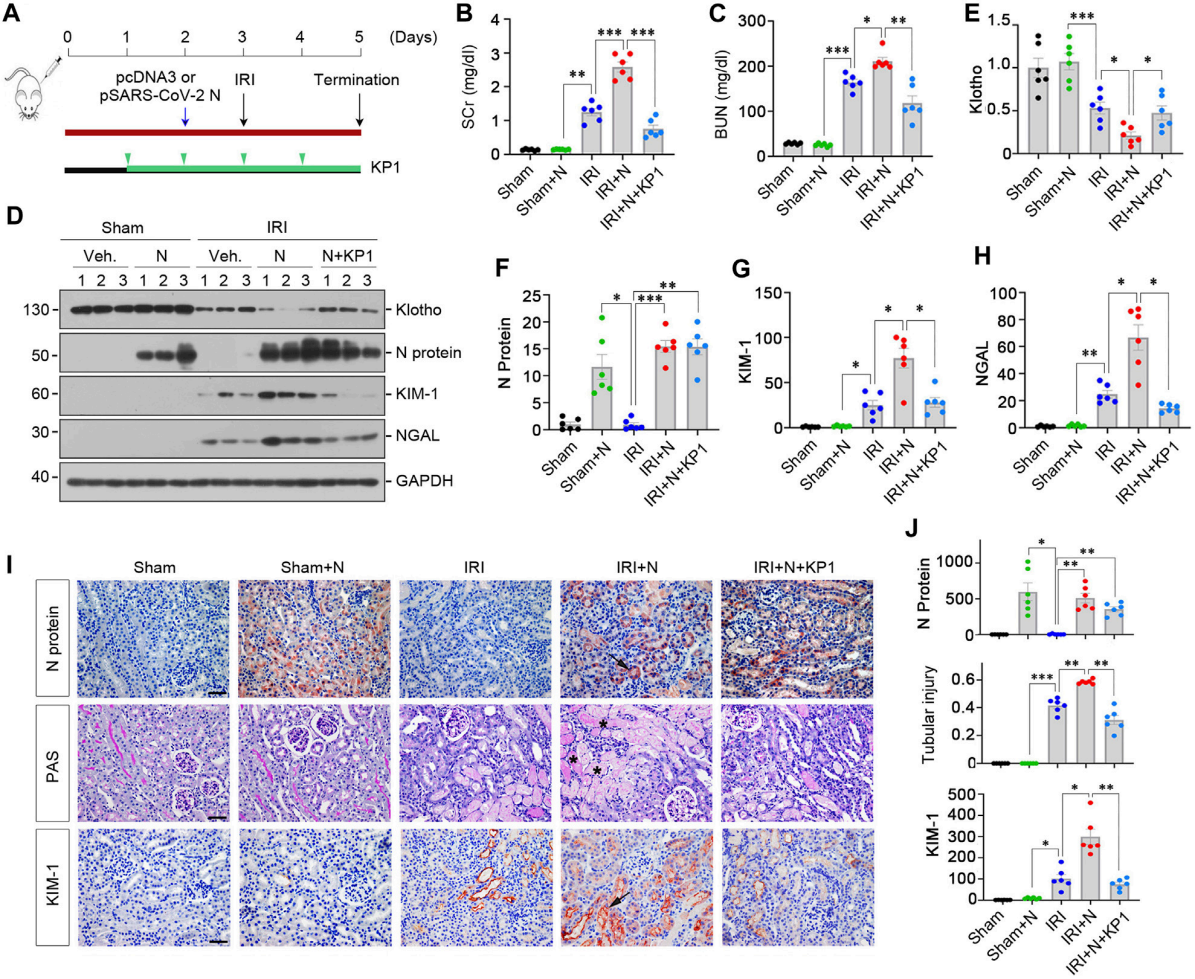
KP1 ameliorates acute kidney injury induced by SARS-CoV-2 N protein and ischemia-reperfusion injury *in vivo*. **(A)** Experimental design. The blue arrow indicates the timing of injecting pcDNA3 empty vector or pSARS-CoV-2 N Protein plasmid (N). The green arrowheads indicate the timing of injecting KP1 at the concentration of 1 mg/day/kg. The black arrow indicates the timing of IRI. **(B, C)** Graphic presentations show SCr and BUN levels in different groups as indicated. ^*^ *p* < 0.05, ^**^ *p* < 0.01, ^***^ *p* < 0.001 (*n* = 6). **(D–H)** Representative Western blot **(D)** and quantitative data show renal protein levels of Klotho **(E)**, N protein **(F)**, KIM-1 **(G)** and NGAL **(H)**. ^*^ *p* < 0.05, ^**^ *p* < 0.01, ^***^ *p* < 0.001 (*n* = 6). **(I, J)** Representative micrographs of Periodic acid-Schiff (PAS) staining and immunochemical staining for N protein and KIM-1 are presented. Arrows indicate positive staining. Scale bar, 50 µm. Semi-quantification data are presented in Panel **(J)**. ^*^ *p* < 0.05, ^**^ *p* < 0.01, ^***^ *p* < 0.001 (*n* = 6).

We further examined the effect of SARS-CoV-2 N protein on the evolution and severity of ischemic AKI. As shown in Figure 6A, mice were subjected to ischemia-reperfusion injury (IRI) 1 day after injection with pSARS-CoV-2 N plasmid. Another group of mice were injected with KP1 daily beginning 2 days prior to IRI (Figure 6A). As shown in Figures 6B, C, SCr and BUN levels elevated at 2 days after IRI, and overexpression of N protein further aggravated renal dysfunction, whereas KP1 decreased SCr and BUN levels (Figures 6B, C). Western blot analyses revealed that the expression of Klotho was downregulated in the kidneys of IRI mice, and overexpressed SARS-CoV-2 N protein accelerated the loss of Klotho, but KP1 partially restored Klotho expression (Figures 6D, E). Furthermore, N protein aggravated KIM-1 and NGAL induction in IRI mice, whereas KP1 inhibited their expression (Figures 6D–H). Similar results were obtained when kidney sections were immunostained for N protein and KIM-1 (Figures 6I, J). We also assessed the renal pathological lesions by Periodic acid-Schiff (PAS) staining. As shown by Figures 6I, J, severe tubular cell loss was evident in IRI kidney, whereas KP1 treatment mitigated these lesions.

**FIGURE 7:**
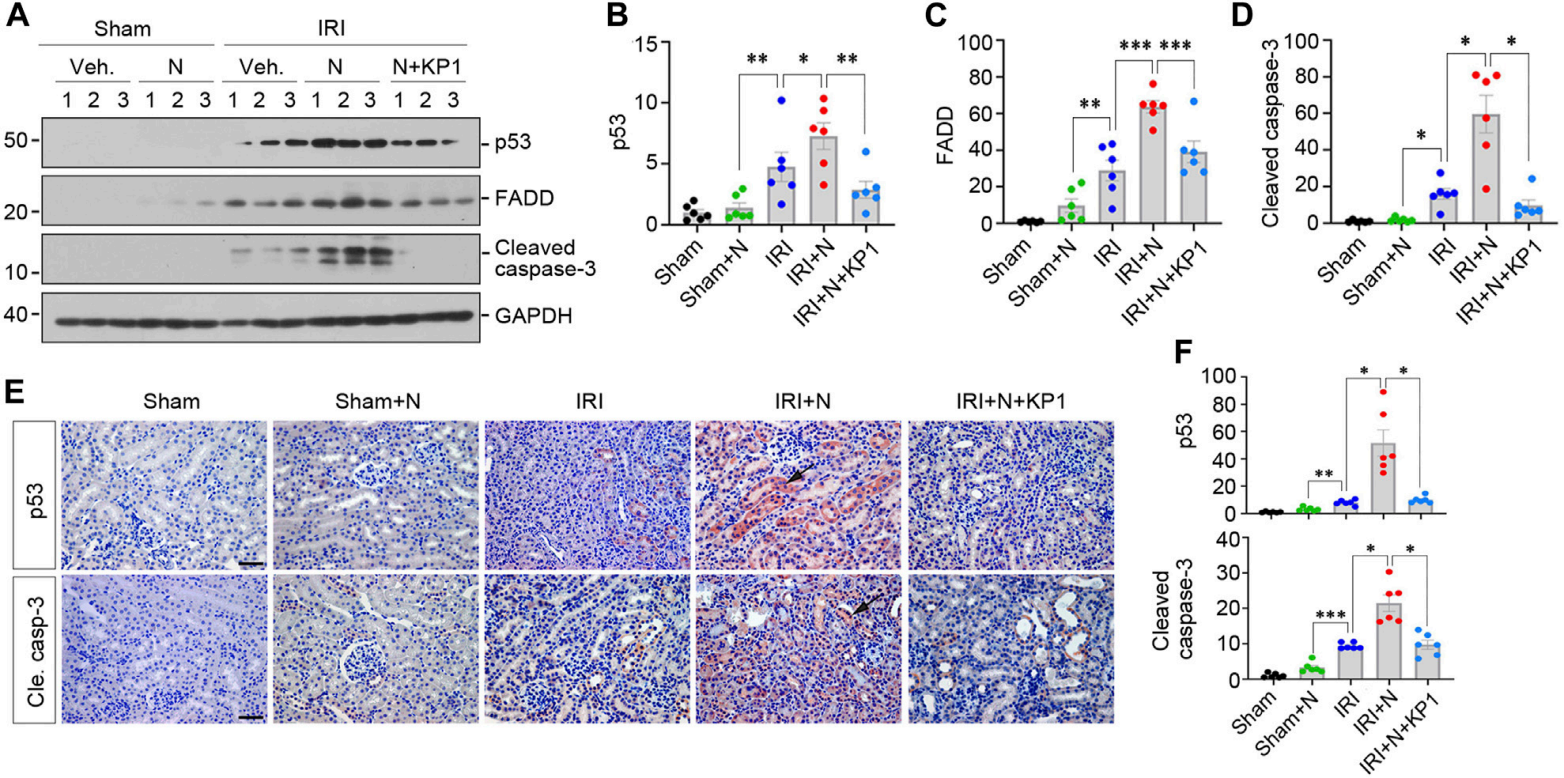
KP1 inhibits renal expression of apoptosis-related proteins induced by SARS-CoV-2 N protein and ischemia-reperfusion injury *in vivo*. **(A–D)** Representative Western blot **(A)** and quantitative data show the renal apoptosis-related proteins including p53 **(B)**, FADD **(C)** and cleaved caspase-3 **(D)**. ^*^ *p* < 0.05, ^**^ *p* < 0.01, ^***^ *p* < 0.001 (*n* = 6). **(E, F)** Representative micrographs of immunohistochemical staining for p53 and cleaved caspase-3 **(E)** and quantitative data **(F)** are presented. Arrows indicate positive staining. Scale bar, 50 μm. ^*^ *p* < 0.05, ^**^ *p* < 0.01, ^***^ *p* < 0.001 (*n* = 6).

We further examined the expression of apoptosis-related proteins, including p53, FADD and cleaved caspase-3. As shown in Figures 7A–D, overexpression of N protein in IRI mice aggravated renal expression of p53, FADD and cleaved caspase-3. However, treatment with KP1 reduced these apoptosis-related proteins. Immunostaining for p53 and cleaved caspase-3 gave rise to similar results (Figures 7E, F). Taken together, these results suggest that SARS-CoV-2 N protein exacerbated AKI induced by IRI, which can be alleviated by KP1.

## 4 Discussion

Aging and preexisting medical conditions are two major risk factors leading to poor outcome and high in-hospital mortality in COVID-9 patients; however, the underlying mechanism between this connection was poorly understood. In the present study, we demonstrate that Klotho deficiency is a common characteristic feature shared in the elderly and those with various medical conditions such as diabetes, hypertension, CKD and cardiovascular disease (Nagai et al., 2000; Kuro-o, 2011; Navarro-González et al., 2014; Neyra et al., 2020). We further show that mutant *Kl/Kl* mice are vulnerable to the expression of SARS-CoV-2 N protein and develop pathological lesions resembling AKI, suggesting that loss of Klotho sensitizes mice to kidney injury caused by SARS-CoV-2. We finally unveil that KP1, a recently discovered Klotho-derived peptide, recapitulates renal protection of Klotho and ameliorates AKI induced by SARS-CoV-2 N protein overexpression. These findings for the first time illustrate that Klotho deficiency is a key determinant of developing COVID-19-associated AKI. Our studies also suggest that delivery of KP1, a small peptide that can be made chemically and cost-effectively, could be an effective strategy to protect kidneys and alleviate AKI in COVID-19 patients.

Overwhelming data from COVID-19 pandemic indicate that the elderly are vulnerable to developing severe COVID-19 with devastating and lethal outcome after SARS-CoV-2 infection (Chen et al., 2021; Wong et al., 2023). The COVID-19 patients with preexisting CKD also exhibits a high burden of comorbidities and multi-morbidity, and higher risk mortality, especially in those who received kidney replacement therapy (Chung et al., 2021; Dashtban et al., 2022). However, such a worsened outcome in these patients appears not due to an increased susceptibility to virus infection *per se*, as angiotensin-converting enzyme 2 (ACE2), the receptor for SARS-Cov-2 infection, is not upregulated in these individuals (Maksimowski et al., 2020). These observations underscore that the N protein, which is essential for causing cell injury, could act differently at distinct settings after SARS-Cov-2 infection. As both aging and CKD have a common feature of Klotho deficiency (Figure 1), this raises the possibility that the state of Klotho deficiency may predetermine the response and fate of individuals to the SARS-Cov-2 infection. Indeed, when the N protein is expressed in *Kl/Kl* mutant mice, they are much more vulnerable than wild type counterparts to N protein expression and develop worsened kidney lesions characterized by an increased expression of KIM-1, NGAL, p53 and FADD (Figure 2). At this stage, how exactly Klotho protects kidney from developing AKI in COVID-19 patients remains unknown, but it could be related to its ability to regulate several signaling pathways. In this regard, Klotho has been shown to inhibit TGF-β signaling and renin-angiotensin system (Zhou et al., 2013; Zhou et al., 2015), both of them are implicated in the evolution of AKI. Furthermore, Klotho could also attenuate ischemic injury and impede AKI progression to CKD by upregulating autophagy (Shi et al., 2016).

Despite some controversies in the literature, increasing evidence alludes that kidney is one of the target organs of SARS-CoV-2 in COVID-19 patients. Kidney tubular cells express abundant ACE2, the membrane receptor essential for SARS-CoV-2 entry into the cell. SARS-CoV-2 can infect kidney cells directly, as demonstrated by RT-PCR (Braun et al., 2020; Hanley et al., 2020; Remmelink et al., 2020), *in situ* hybridization (Diao et al., 2021), immunohistochemical staining (Bradley et al., 2020; Schurink et al., 2020) and immunofluorescence staining (Su et al., 2020). In addition, SARS-CoV-2 may also induce kidney damage indirectly by triggering inflammatory cytokine storm and immune disorders (Lin et al., 2023). The typical clinical manifestations of kidney injury caused by SARS-CoV-2 are decreased renal function, tubular cell death, proteinuria, hematuria, and renal infarction. At the cellular level, SARS-CoV-2 can provoke damage on almost all types of renal parenchymal cells, including tubular epithelial cells, podocytes, mesangial cells and endothelial cells (Lin et al., 2023). As tubular epithelial cells are the epicenter of kidney injury involved in AKI, we specifically investigated their responses to SARS-CoV-2 N protein expression. Overexpression of SARS-Cov-2 N protein alone causes tubular HK-2 cells to undergo a spectrum of changes (Figure 3), characterized by apoptosis, cellular senescence and partial epithelial-mesenchymal transition (pEMT). Such findings are supported by an earlier report that SARS-CoV-2 N protein is present in the cytoplasm of proximal tubules and SARS-CoV-2 infection is associated with tubulointerstitial fibrosis as shown by single-nucleus RNA sequencing (Jansen et al., 2022). Therefore, it is conceivable to speculate that the expression of N protein after SARS-CoV-2 infection not only causes tubular cell apoptosis leading to AKI but also induces cell senescence and fibrotic responses, possibly promoting its progression to CKD.

While tremendous effort and progress have been made in preventing SARS-CoV-2 infection, there are few options to ameliorate organ dysfunction and injury after COVID-19 develops. Up to date, there is no sufficient and effective treatment option to prevent and protect against COVID-19-asociated AKI in patients (Jansen et al., 2022). Drugs such as corticosteroids, tocilizumab and remdesivir exhibit no significant benefit for kidney or mortality in clinical studies (Kumar et al., 2022; Burger et al., 2023; Grundeis et al., 2023). Kidney replacement therapy (KRT) is currently the only treatment for severe AKI. In this context, it is paramount to develop therapeutics to combat AKI in COVID-19 patients. Given that Klotho deficiency predisposes mice to developing AKI after SARS-Cov-2 N protein expression (Figure 2), it is plausible that delivery of exogenous Klotho can mitigate tubular cell injury and apoptosis triggered by SARS-Cov-2 N protein *in vitro* (Figure 4). However, as a large membranous protein with a complex structure, it is difficult and costly to produce Klotho in large quantity to meet clinical applications. We recently discovered KP1, a small Klotho-derived peptide that recapitulates the function of Klotho by targeting and inhibiting TGF-β signaling (Yuan et al., 2022). In the present study, we show that KP1 alleviates tubular injury and apoptosis induced by SARS-CoV-2 N protein expression *in vitro* and *in vivo* (Figures 4, 6). Notably, the efficacy of KP1 in mitigating tubular injury triggered by N protein is comparable to Klotho itself (Figure 4). The mechanism by which KP1 ameliorates AKI induced by SARS-CoV-2 N protein expression remains to be delineated, but it could be related to its ability to block TGF-β/Smad signaling. This speculation is strengthened by a recent study that SARS-CoV-2 N protein could induce AKI via triggering TGF-β/Smad3-dependent G1 cell cycle arrest (Wang et al., 2022). Taken together, the results in the present study suggest that KP1 may hold the potential as a promising remedy in preventing and treating COVID-19-related AKI. However, whether KP1 remains effective after AKI has been developed, a situation closer to the real one in a hospital, deserves further investigation.

In summary, we have shown in this study that Klotho deficiency, a common feature shared by aging and various preexisting medical conditions, is a key determinant of developing AKI in COVID-19 patients. As such, mice deficient of Klotho are sensitized to develop kidney lesions resembling AKI upon expression of SARS-CoV-2 N protein. Furthermore, we demonstrate that KP1, a recently discovered Klotho-derived peptide, effectively alleviates tubular cell injury and apoptosis induced by SARS-CoV-2 N protein after ischemic injury. Although many issues remain to be resolved, the present study paves a new avenue for developing an effective therapeutic for combating kidney injury in COVID-19 patients.

## Data Availability

The raw data supporting the conclusion of this article will be made available by the authors, without undue reservation.

## Abbreviations

AKI, acute kidney injury; CKD, chronic kidney disease; COVID-19, coronavirus disease 2019; FADD, FAS-associated protein with death domain; KIM-1, kidney injury molecule-1; NGAL, neutrophil gelatinase-associated lipocalin.
